# Efficient and scalable gene delivery method with easily generated cationic carbon dots

**DOI:** 10.1186/s12575-024-00232-7

**Published:** 2024-03-08

**Authors:** Manuel Algarra, Elena Gonzalez-Muñoz

**Affiliations:** 1 INAMAT2—Institute for Advanced Materials and Mathematics, Department of Science, Public University of Navarra, 31006 Pamplona, Spain; 2 Instituto de Investigación Biomédica de Málaga y Plataforma en Nanomedicina (IBIMA plataforma BIONAND) C/ Severo Ochoa, 35 Málaga, Spain; 3 Departamento de Biología Celular Genética y Fisiología, Universidad de Málaga, 29071 Málaga, Spain

**Keywords:** Carbon dots, Polyethyleneimine (PEI), Cell transfection, Gene delivery, Cell transduction

## Abstract

Gene delivery is a complex process with several challenges when attempting to incorporate genetic material efficiently and safely into target cells. Some of the key challenges include not only efficient cellular uptake and endosomal escape to ensure that the genetic material can exert its effect but also minimizing the toxicity of the delivery system, which is vital for safe gene delivery. Of importance, if gene delivery systems are intended for biomedical applications or clinical use, they must be scalable and easy and affordable to manufacture to meet the demand. Here, we show an efficient gene delivery method using a combination of carbon dots coated by PEI through electrostatic binding to easily generate cationic carbon dots. We show a biofunctional approach to generate optimal cationic carbon dots (CCDs) that can be scaled up to meet specific transfection demands. CCDs improve cell viability and increase transfection efficiency four times over the standard of PEI polyplexes. Generated CCDs enabled the challenging transfection protocol to produce retroviral vectors via cell cotransfection of three different plasmids into packing cells, showing not only high efficiency but also functionality of the gene delivery, tested as the capacity to produce infective retroviral particles.

**Supplementary Information:**

The online version contains supplementary material available at 10.1186/s12575-024-00232-7.

## Introduction

Cell transfection efficiency is a limiting factor for a number of biotechnology assays that require high rates of gene transfer. Gene delivery strategies are generally classified according to their need for integration into the cell genome to be expressed, which depends on the nature of the exogenous DNA constructor (integrative or non-integrative) and on the nature of the vector that allows entrance into the target cell (viral or non-viral). These characteristics will determine the efficiency, reproducibility, and safety (referring to the magnitude of potential harm produced to the cell) of the method. Due to their exceptional infectivity, virus-based vectors typically exhibit excellent gene transfection capabilities [1, 2], although they can potentially induce cytopathic effects, or random mutagenesis when using integrative constructors, and their use tends to be avoided when seeking biomedical applications.

For the introduction of non-viral vectors, it is necessary to use a delivery method that allows entry of the DNA vector into the cell for its expression. Despite their biological safety advantages for cells, non-viral vectors have not been used much due to their relatively low efficiency of gene delivery [3]. There are different methodologies that can be classified into physical and chemical/carrier-based methods.

Physical methods include electroporation, particle bombardment, sonoporation, photoporation, and magnetofection, which utilize electrical pulses, force, sound, light, and magnetic fields to increase the permeability of cell membranes to allow genetic materials to enter cells [4, 5]. However, these physical methods often cause a high rate of cell death during the process and greatly affect their phenotype, potentially affecting subsequent processes to be analyzed and then increasing the risk of artifactual results although there has been relevant progress with specific strategies as photoporation or electroporation among others [6, 7].

The alternative is to employ synthetic or natural biocompatible materials as carriers to deliver genetic materials that are able to enter cells by endocytosis. The most extended chemical/carrier-based vectors are liposomes and polymers. Liposome-based non-viral vectors use liposomes to facilitate gene delivery by the formation of lipoplexes. Lipoplexes form spontaneously when negatively charged DNA contacts positively charged liposomes and great advanced has been made to improve transfection efficiency and overcome toxicity of cationic lipids and even commercial formulation based on lipofectamine reagents are commonly used in cell biology with different degree of efficacy and toxicity [8]. Polymer-based non-viral vectors use polymers to interact with DNA to form polyplexes [9]. Polyethyleneimine (PEI) is the cationic polymer most frequently used as a transfection reagent. It is positively charged and can form complexes with negatively charged nucleic acids, such as DNA or RNA. These complexes can efficiently deliver nucleic acids into cells by interacting with the cell membrane and facilitating their internalization. Several forms of PEI, considering its molecular weight and structure, have been used for gene delivery. The most common is 25 kDa PEI, either with a branched or linear structure, and the linear 25 kDa form is the favorite among a majority of cell biologists, suggesting a better balance between DNA cell transfection efficiency and acceptable cell toxicity [10].

Carbon dots (CDs), also known as carbon quantum dots, are nanoscale carbon-based materials with unique optical and chemical properties [11]. They are primarily generated from carbonaceous sources and have undergone substantial research for a variety of uses, such as gene transfection, medication administration, and cell imaging. CDs can be functionalized to enhance their interactions with cells and have strong biocompatibility [12, 13].

It has been reported that when CD has positive charges on the surface coming from amine groups incorporated during their synthesis, they can bind to negatively charged DNA by electrostatic interactions to form CD-DNA complexes that can then penetrate cell membranes via mechanisms such as phagocytosis, endocytosis, and micropinocytosis [14–17]. However, gene delivery efficiency has not been extensively analyzed in these reports, and two main strategies have been used to further improve the binding efficiency, obtaining more positively charged CDs. Either (1) using positively charged molecules as precursors, such as polyethyleneimine (PEI), polyethylene diamine (PAMAM), chitosan, poly-L-lysin (PLL), and pentaethylenehexamine (PEHA), for the synthesis of CDs [18] or (2) through a two-step method where CDs containing -COOH and hydroxyl (-OH) groups on their surface are first generated through different synthetic approaches, such as hydrothermal, pyrolytic or microwave-assisted approaches, which results in a high density of functional groups, and then, high density -NH2 compounds are grafted on the surface of the CDs by either amide reaction or electrostatic attraction, generating positively charged cationic CDs (CCDs) [19].

Even if electrostatic interaction is a relatively weak force, the assembly of CCDs using this approach has the advantage of simplicity over the rest of the methods described; however, its use has been limited to a few studies using PEI as a capping agent but—to the best of our knowledge—not related to gene delivery but to the detection of trace Cr in environmental water samples [20] or for efficient targeted cancer cell imaging [21].

As mentioned, in some studies, a combination of carbon dots and PEI has been employed for cell transfection, reporting a certain degree of efficiency for gene delivery (reviewed in [22]), with very limited studies showing significant improvement over commercial lipoplexes or polyplexes [23]. The reported strategies for CCD generation have been focused on a one-step method using branched PEI together with a carbon precursor molecule for the synthesis of CCDs, and only one reported study where a variant form of PEI (alkyl-PEI, 2 kDa) was grafted after CD synthesis by an amide reaction [24].

Here, we describe the generation of an efficient gene delivery method based on CCDs coated with linear PEI by electrostatic interactions. This method not only greatly improves cell transfection over commercial PEI polyplexes but also allows simple and quick preparation of the CCDs, enabling their use in a broad range of biological applications without dependence on specialized chemical synthesis but also allows combination with different ratios of PEI that enable optimization for specific plasmids/genes of interest, as the optimal DNA/PEI interaction depends on the nitrogen/phosphate (N/P) ratio, which depends on the length of the plasmid/DNA [25], and for specific cell types [26].

Gene delivery is especially challenging when either the total amount of DNA is high (high size vectors) or different plasmids/DNA need to be transfected simultaneously to achieve a specific biological aim. Standard retroviral or lentiviral vector production constitutes an ideal model of a demanding assay to test gene delivery efficiency, as it needs three different high size vectors to be delivered into the packing cell for proper viral particle production, and the functional analysis of efficient exogenous gene expression can be readily analyzed by cell transduction measurement, i.e., the capacity of produced viral particles secreted to the supernatant to infect target cells. We show that generated CCDs can efficiently be used to produce retroviral vectors encoding bicistronic constructors expressing both GFP and Sox15 (a cell reprogramming factor), constituting an ideal resource for complex transfection applications.

## Material and methods

### Materials

Citric acid (≥ 99.5%) was purchased from Thermo Fisher (Kandel, Germany), and Thermof PEHA (P98%), 3-[4,5-dimethylthiazol-2yl]-2,5-diphenyl tetrazolium bromide (MTT), phosphate-buffered saline (PBS), and Triton-X-100 were obtained from Sigma-Aldrich (St. Louis, MO, USA). Dialysis tubing with a MWCO of 100–500 Da was obtained from Spectrum Labs, Inc. (Rancho Dominguez, CA). Milli-Q water (18.2 MΩ, filtered with filter pore size 0.22 µM) was obtained from Millipore and used as the solvent in the preparation of CDs. Dimethyl sulfoxide (DMSO) was supplied by Acros Organics B.B.V.A. (Verona, Italy). Polyethylenimine, Linear, MW 25000, Transfection Grade (PEI 25 K™) was obtained from Polysciences Inc (Cat#23966) and a solution at 1 mg/mL was prepared in Milli-Q water and balanced to pH7. Dulbecco’s modified Eagle’s medium (DMEM), fetal bovine serum (FBS), L-glutamine, and penicillin/streptomycin solution were obtained from Gibco. All chemicals were used without further treatment. The bicistronic retroviral vector pMX-GFP-Sox15 has been previously constructed in our laboratory [27, 28], and packaging proteins encoding plasmid pCMV-GAG-POL and envelope protein encoding plasmid pCMV-VSV and 6050 bp plasmids were obtained from Addgene repositories (Addgene Cat#14887 and Addgene Cat#8454)).

### Preparation of carbon dots

Citric acid was used as a precursor to generate CDs using a hydrothermal approach. First, an aqueous dissolution of citric acid (50 g/L) was transferred into a Teflon-lined beaker and placed inside a steel reactor. Then, it was heated in an oven at 200 ºC and left for 4 h. The recipient was then allowed to cool to room temperature (25ºC). Before the reaction products were centrifuged to remove any insoluble materials. The solution was then subjected to dialysis for 24 h to clean up the carbon nanoparticles (CDs).

### Optical characterization of CDs

UV/vis absorption spectra of CDs were obtained using a Jasco V-730 UV–Vis between 200–600 nm. PhotonTechnologyInternational (PTI)Inc. A QuantaMaster40 spectrofluorometer equipped with a 75 W continuous xenon arc lamp was used. An ASOC-10 USB interface FeliX GX software was used for fluorescence data acquisition, and the hardware was controlled for all system configurations. The slit widths for excitation and emission were both 2 nm. All optical measurements were performed in a 10 mm quartz cell at room temperature (25 °C).

### Dynamic light scattering (DLS)

An aqueous suspension with a CD or CCD concentration of 50 mg·mL^−1^ was prepared to obtain information on the size distribution and zeta potential measurements using dynamic light scattering (DLS, Malvern Zetasizer Nano-ZS90). The measurements were performed on a cell type: ZEN0118-low volume disposable sizing cuvette, setting 2.420 as the refractive index with 173° backscatter (NIBS default) as the angle of detection. The measurement duration was set as automatic, and three was set as the number of measurements. A general-purpose analysis model was chosen (normal resolution).

### Transmission electron microscopy

Transmission electron microscopy (FEI Tecnai G2 Twin microscope) operated at an accelerating voltage of 100 kV was employed to assess particle shape and size. A droplet of an aqueous suspension containing the CDs and CCDs was placed onto a carbon-coated copper grid, and the water was allowed to evaporate for TEM analysis.

### Agarose gel retardation assay

Agarose gel electrophoresis was conducted to study CCD binding with DNA. Briefly, CCD aqueous solutions prepared at a 20:3 (ug CD: ug PEI) ratio were incubated at different mass ratios of CCDs (100: 1, 40:1, 20:1, 10:1) with DNA. The as-obtained solutions were incubated at room temperature for 30 min, followed by agarose gel electrophoresis analysis in TAE buffer for 60 min at 90 V. The results were observed using BioRad-image lab software with UV light. The aforementioned DNA solutions and PEI:DNA (3:1 mass ratio) were applied as controls.

### Cell line culture conditions, cell transfection and transduction

HEK293T cell line was obtained from Sigma Aldridch (#12022001). Primary somatic stromal cell lines MenSC (also called MnSC) were previously generated after donor informed consent and with clearances from the bioethical committee and Review Board of the National Research Ethics Service (#PR-03–2018) [27]. HEK293T cells were cultured with high glucose in DMEM (with 10% FBS, 1% glutamine and 1% penicillin–streptomycin). MenSC cells were cultured in DMEM-F12 containing 10%FBS, 1xNEAA, 1xL-Glutamine, penicillin and streptomycin as previously described [27, 28]. We followed standard transient transfection using the PEI method as previously described [28] or generated CCDs as described in the results "Effect of CDs on cell viability" section. For retroviral production, HEK293T cells were plated at 70% cell confluence in 100 mm dishes. After 24 h, cells were transfected with a transgene encoding the pMX viral vector, Gag-Pol vector and VSV-G plasmid using either the polyethylenimine method or generated CCD particles. The supernatant was collected 24 h posttransfection and filtered through 45-mm pore size filters. Cell transduction was performed on HEK293T or MenSC cells using 2 mL of unconcentrated viral supernatant to transduce 100,000 cells in the presence of 4 μg/mL polybrene.

### Cell viability assay

Cell viability was evaluated in HEK293T cells by MTT assay. Briefly, cells were plated at a density of 1 × 10^4^ cells/well in a 96-well plate at 37 °C in a 5% CO_2_ atmosphere (200 μL per well, number of replicates = 5). After 24 h of culture, the cell culture medium was replaced with fresh medium containing CCDs (or PEI) at different concentrations. After 24 h, the supernatant was replaced by 200 μL/well of fresh medium with 3-[4,5-dimethylthiazol-2-yl]-2,5-diphenyl tetrazolium bromide (MTT) (0.5 mg⋅mL^−1^). After 2 h of incubation at 37 °C and 5% CO_2_, the medium was removed, the formazan crystals were solubilized with 200 μL of DMSO, and the solution was vigorously mixed to dissolve the reacted dye. The absorbance of each well [Abs]well was read on a microplate reader (Dynatech MR7000 instruments) at 550 nm. The relative cell viability (%) and its error related to negative control wells containing cell culture medium without nanoparticles and positive control wells where Triton X-100 was added to the cells were calculated by the following equations:

$$RCV(\%) = (([Abs]test- [Abs]Pos.Ctrl.) / ([Abs]Neg. Ctrl-[Abs]Pos. Ctrl)) x100$$

$$\mathrm{Error }(\mathrm{\%}) =\mathrm{RCVtest x SQRT}\left[{\left(\mathrm{\sigma test}/\left[{\text{Abs}}\right]{\text{test}}\right)}^{2}+{\left(\mathrm{\sigma tes}/\left[{\text{Abs}}\right]{\text{test}}\right)}^{2}\right]$$

where σ is the standard deviation.

### Flow cytometry

HEK293T cells were harvested at 300xg for 5 min, fixed in 4% v/v formaldehyde for 15 min, centrifuged at 500 g for 5 min, and finally resuspended in 0.3 mL of PBS. The percentage of GFP-positive cells was assessed using a flow cytometer (Beckman Coulter Gallios). The 488 nm laser was used for GFP measurement in the FITC-A detector. A total of 100,000 events were analyzed in every sample. First, side scatter height (SSC-H) *vs* forward scatter area (FSC-A) and FSC-A vs forward scatter height (FSC-H) density plots were used to gate the individual cell population. Then, the GFP-positive population was assessed in a GFP fluorescein isothiocyanate (FITC) vs FSC-A density plot and in a GFP FITC histogram using cells transfected with only GagPol and VSV vectors (no GFP encoding vector) as a negative control. Data were analyzed using Kaluza Beckman Coulter software (Beckman Coulter).

## Results and discussion

### CD synthesis and optical property characterization

**Fig. 1:**
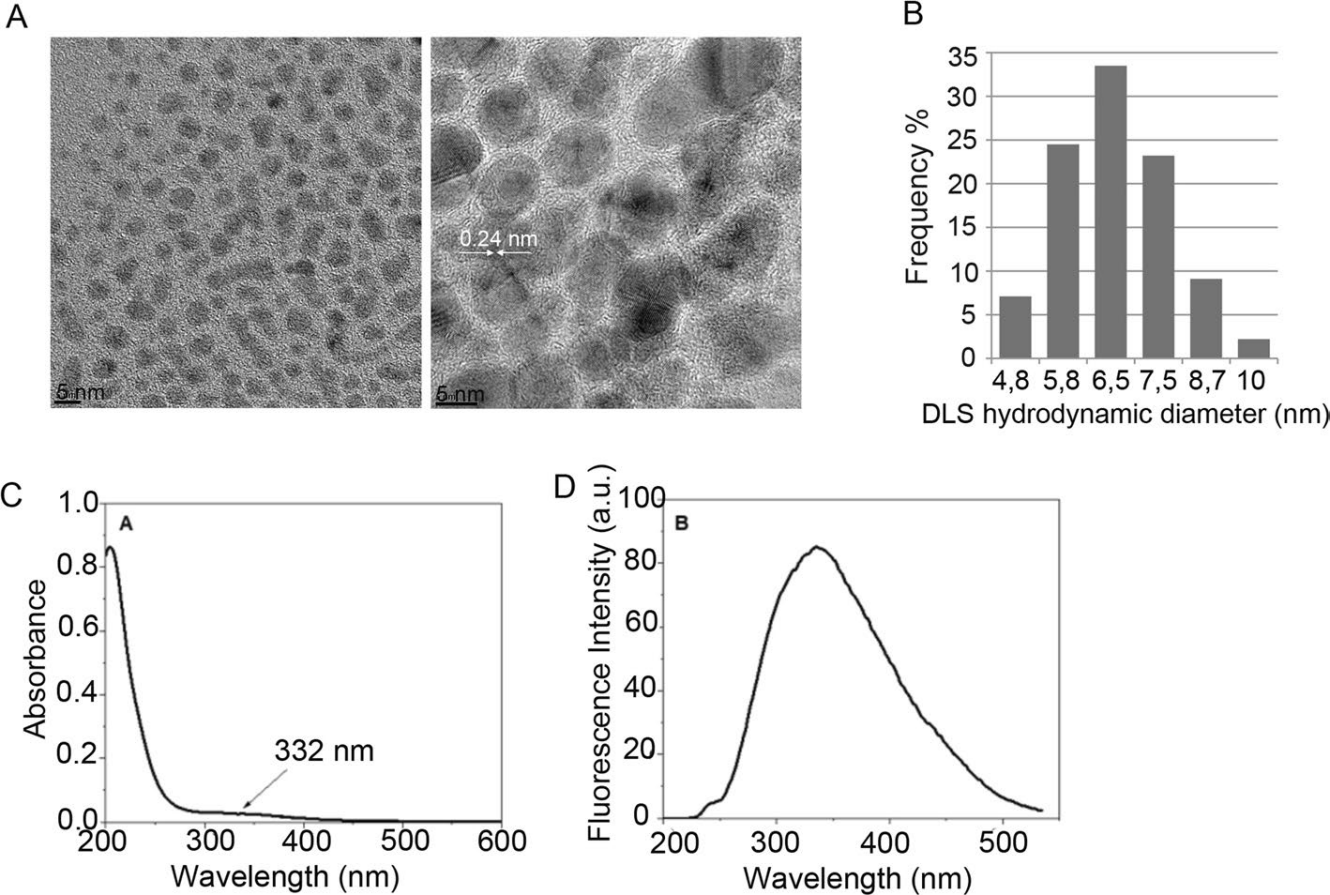
**A** HR-TEM images of the CDs generated after the reaction of citric acid at 200 °C at two magnifications (scale bar 5 nm). **B** Histogram frequency of DLS measurements of the mean hydrodynamic diameter (nm) found in CD suspensions. **C** UV/vis and **D** fluorescence spectra of the CDs generated from citric acid

Carbon-based nanoparticles (CDs) were generated hydrothermally as described in "Preparation of carbon dots" section. CDs show a spherical and very uniform morphology, with an average size of less than 5 nm (Fig. 1A) and a hydrodynamic diameter below 10 nm (Fig. 1B). This synthetic method allows the nanoparticles to have the typical graphitic structure, as shown in Fig. 1A. Layers of sp2-hybridized hexagonal carbon rings make up graphite. The HR-TEM images show a distinct 0.24 nm lattice fringe, revealing that the CDs produced from citric acid likewise have a graphite origin [11, 29].

The optical properties of the CDs were further examined. The UV/vis spectrum of CDs shows a small band at 332 nm (Fig. 1C) that can be attributed mainly to the n-p* electronic transition of the surface carbonyl groups (C = O) [30]. Photoluminescence (PL) spectra are one of the most crucial properties of CDs in biomedical applications, although some applications, such as cell tracking, require fluorescence emission by CDs. Many cell biology applications of gene delivery use fluorescent reporter genes or engineered fluorescent fusion proteins to further follow the effects of exogenous gene constructors on cell function, and thus, CD PL spectra should not interfere with the detection of widely used fluorescent reporter genes, such as GFP, YFP or RFP*.*

In this study, we used a retroviral bicistronic vector encoding GFP and the oocyte-enriched factor Sox15 for cell reprogramming after overexpression on target cells [27]. Detection of the GFP reporter gene is used for tracking cells expressing the reprogramming factor Sox15 and thus the reprogramming process over time. The fluorescence emission spectra of CDs showed a PL band at 340 nm (Fig. 1D), a typical behavior when CDs are obtained from citric acid at 200 ºC [30], thus confirming that it does not interfere with the detection of the GFP emission peak at ~ 510 nm [28].

### Biofunctional approach for cation carbon dot (CCD) generation

#### Effect of CDs on cell viability

To generate CCDs by electrostatic binding of PEI, we incubated different amounts of CDs with PEI with agitation for 3 h at 25 °C.

We used a biofunctional approach to establish the CD:PEI ratio for CCD generation.

**Fig. 2:**
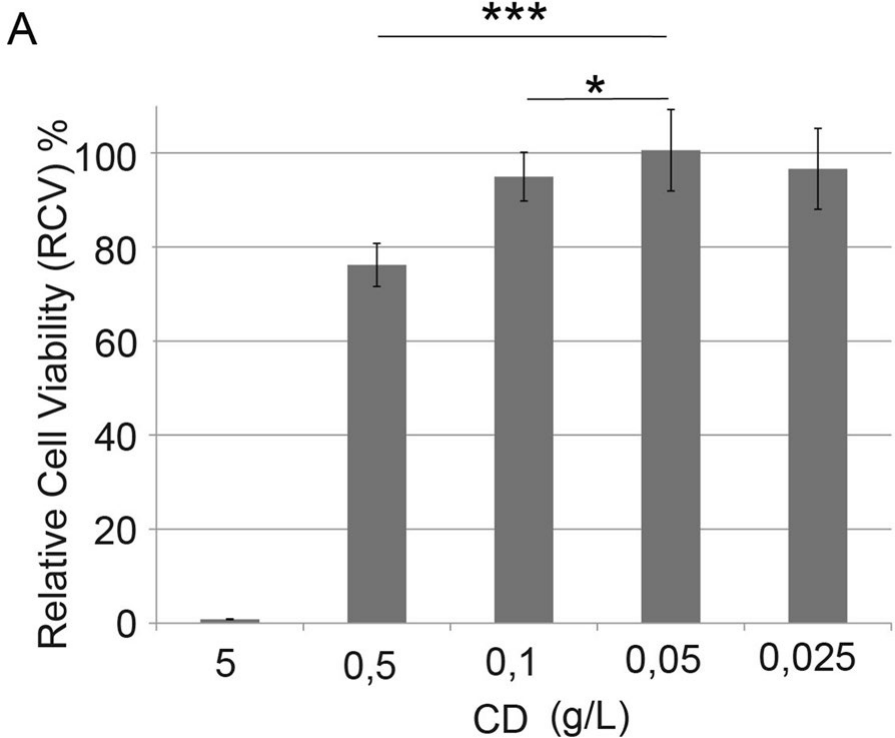
Relative cell viability of the HEK293T cell line after 24 h of incubation with CDs at different concentrations. (*n* = 5, mean values ± SD. Student’s t-test was applied for statistical significance: *P < 0.05 ***P < 0.001)

We first established the optimal concentration of CDs for cell survival using the MTT assay, and 0.05 g/L allowed the highest cell viability (Fig. 2).

#### Optimization of CCD generation by electrostatic PEI binding

Second, we used our previously optimized control parameters of cell transfection via PEI polyplexes using a PEI:DNA mass ratio of 3:1—7.5 of PEI with 2.5 µg of DNA plasmid pMX-per mL of final cell medium per well of a 6-well plate (p6) [27, 28] as the standard starting point for CD:PEI:DNA ratio optimization.

We calculated the optimal amount of CDs to combine with PEI using these two parameters and the optimal amount of CDs for cell survival (50 µg/mL) with the µg of PEI needed in the abovementioned control polyplex transfection (7.5 µg). Therefore, we set our starting test mass ratio CD:PEI: µg as 20:3.

We thus prepared CCDs by mixing different amounts of CDs with a fixed amount of PEI to obtain different ratios: 10:3, 20:3, 40:3, 100:3 and 200:3.

**Fig. 3:**
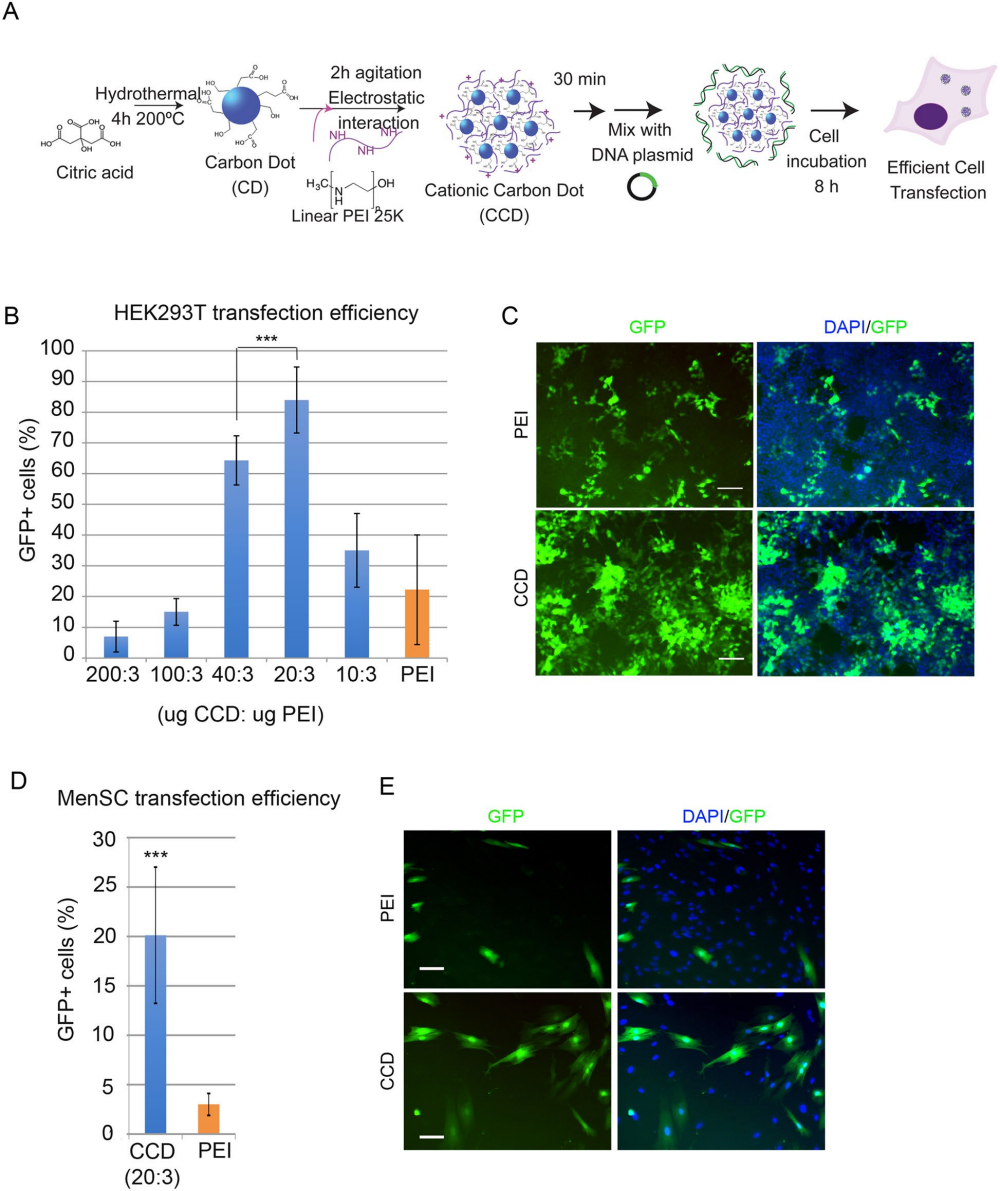
**A** Schematic of CCD preparation and cell transfection **B** Transfection efficiency measured as the % of GFP-positive cells analyzed by flow cytometry of HEK293T cells plated in p6 wells and transfected with CCDs generated by incubation of CDs with PEI at different mass ratios and mixed with a fixed amount of DNA. PEI at a 3:1 mass ratio with DNA was used as a control (orange) (*n* = 7, mean values ± SD. Student’s t-test was applied for statistical significance: ***P < 0.001). **C** Representative fluorescence microscopy images of transfected HEK293T cells with CCDs (20:3 mass ratio condition) or PEI (scale bar = 100 µm). **D** Transfection efficiency measured as the % of GFP-positive cells analyzed by flow cytometry of MenSC cells plated in p6 wells and transfected with CCDs (20:3 mass ratio condition). **E** Representative fluorescence microscopy images of MenSCs transfected cells with CCDs or PEI (scale bar = 100 µm)

After 30 min, we combined electrostatically generated CCDs with plasmid DNA at the described fixed amount (2.5 µg per p6 well) to maintain the PEI:DNA mass ratio at 3:1, vortexed briefly and added them to cell media to test the cell transfection efficiency (Fig. 3A).

We found 20:3:1 as the most efficient gene delivery condition, increasing more than 4 times the “positive transfection standard” PEI polyplex transfection efficiency (Fig. 3) and reaching over 80% GFP-positive cells measured by flow cytometry and visualized by fluorescence microscopy.

To confirm we reached the maximum transfection efficiency, we used different starting concentration of CD showing low cell toxicity (Fig. 2), 100 µg/mL and 25 µg/mL, combined with PEI at different mass ratio CD:PEI: 10:3, 20:3 and 40:3 (Supplementary Table S1), confirming they show lower efficiency than using 50 µg/mL of CD at 20:3 CD:PEI mass ratio. We also assayed to combine different amount of DNA with the CCD generated at the 20:3 CD:PEI mass ratio (Supplementary Table S2) and confirmed 1 µg of plasmid DNA (20:3:1 CD:PEI:DNA mass ratio) as the most efficient condition.

We confirmed the efficiency of CCD transfection also with primary cells. Primary cell cultures often show low transfection rates thus complicating their applications [31]. Mesenchymal cells can be obtained from different origins and present wide biomedical applications, of importance menstrual blood derived stromal cells (MenSCs) have recently emerged as cells with relevant regenerative, reparative, and protective properties of MenSCs and their therapeutic potential is being studied using recombinant protein expression among others technologies, however there are still very limited studies of transfection for gene delivery on MenSCs [32]. We confirmed CCD significantly increases MenSC cell transfection (Fig. 3D,E), opening the venue to assay these particles for different primary cell cultures gene overexpression.

#### CCD size characterization

The appropriate surface charge and particle size of the DNA complexes are crucial for gene delivery. It is commonly accepted that complexes within the size range of 40 ∼ 200 nm may encounter better endocytosis [33]. Below 100 nm, the size of the nanoparticle plays a less important role in possible routes of uptake, as the geometry of different endocytic pathways can readily accommodate small nanoparticles.

**Fig. 4:**
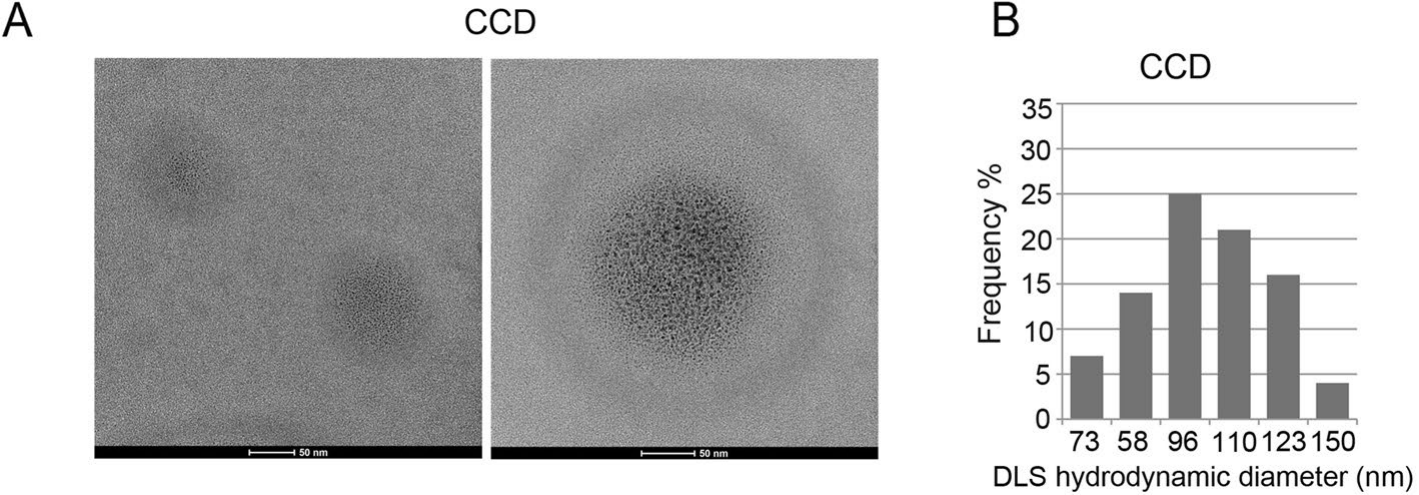
**A** TEM images of a 50 g/L CCD suspension prepared as described by 3 h of mixing CDs with PEI at a 20:3 optimized mass ratio (scale bar 50 nm), 30 min after preparation **B** Histogram frequency of DLS measurements of the mean hydrodynamic diameter (nm) found in CCD suspensions 30 min after preparation

The size of the CCDs 30 min after preparation was measured by TEM and DLS (Fig. 4). While CDs were visualized as single dots less than 10 nm in diameter (Fig. 1), generated CCDs aggregated to form over 50 nm diameter structures (Fig. 4A) with an average DLS diameter of approximately 100 nm (Fig. 4B).

CCD size and DLS diameter slightly increased over time but remained below ∼200 nm 2 h and 8 h after CCD preparation (Supplementary Figures S1A-D) and formed bigger aggregates of ∼ 1 μm after 24 h (Supplementary Figures S1E,F). We confirmed that transfection efficiency inversely correlated with CCD size (Supplementary Figures S1G) and kept 30 min as the optimal time to use for cell transfection.

#### Effect of CCDs on cell viability

**Fig. 5:**
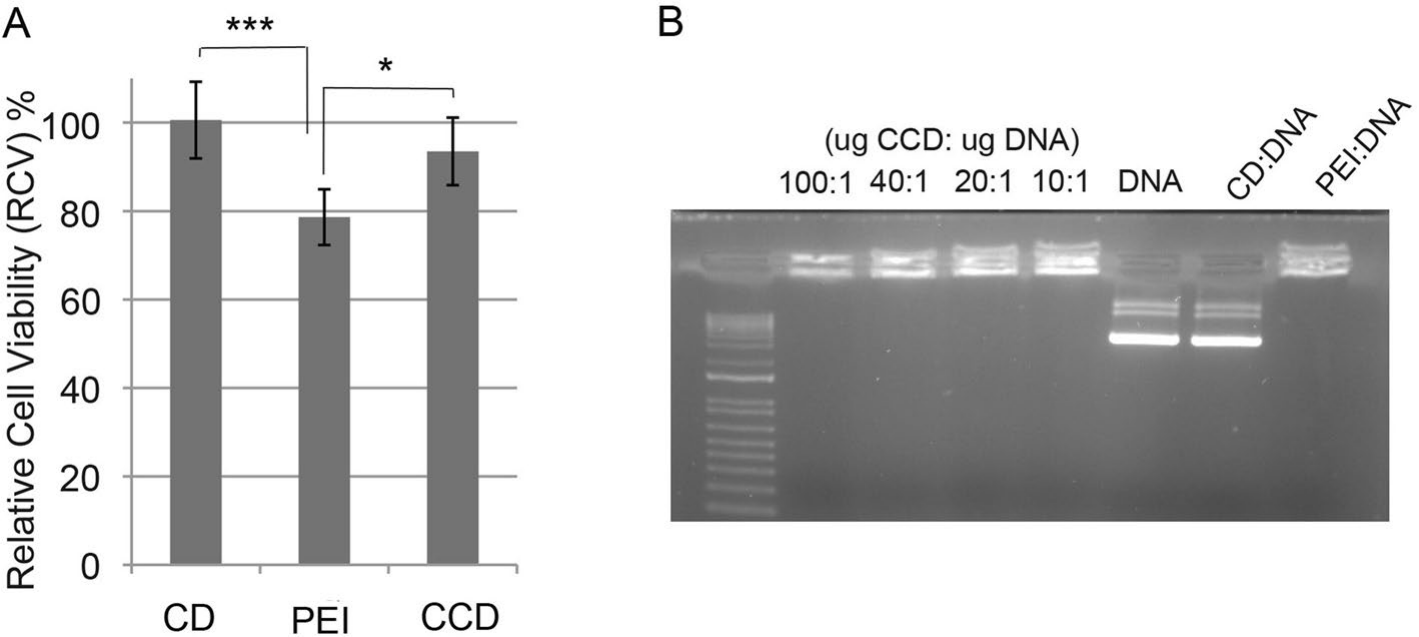
**A** Relative cell viability after 24 h of incubation with CDs at 0,05 g/L, CCDs at a 20:3 CD:PEI mass ratio and PEI at 0,016 g/L using the MTT assay. (*n* = 9, mean values ± SD. Student’s t-test was applied for statistical significance: * *P* < 0.05 *** *P* < 0.001. **B** Agarose gel electrophoresis of the complexes at different mass ratios

We then confirmed that the use of CCDs improved cell viability compared to PEI and, as expected, slightly decreased cell viability compared to naked CDs, as PEI has been widely shown to affect cell survival [34, 35] (Fig. 5A).

We also evaluated the relative cell viability (%RCV) when using CCD generated at different concentrations of CD and CD:PEI mass ratio (Supplementary Table S1), again showing maximum both cell transfection and cell viability with CCD generated with CD:PEI 20:3 mass ratio shown in Fig. 5A.

A gel retardation assay was used to confirm the interaction between the CCDs and DNA. As shown In Fig. 5B, DNA could be completely trapped in the loading slot by both PEI polymers and generated CCDs, while naked CDs were unable to bind DNA that migrated along the lane, similar to unbound DNA.

The zeta potential of the complexes was evaluated and found to increase from − 0.456 mV in CDs to + 18.2 mV in CCDs, confirming their positive charge after electrostatic coating with PEI, which allows efficient interaction with DNA. CCDs present a smaller charge than PEI polyplexes (+ 35 mV), and this effect may explain the decreased damage to cell membranes, thereby reducing the cytotoxicity observed when using CCDs (Fig. 4A) [36–38].

### Evaluation of CCDs for the generation of retroviral particles

We used our adjustable protocol for the generation of CCDs for the challenging generation of retroviral particles through plasmid cotransfection at a different scale to confirm the robustness of the method.

For efficient retroviral production, 100 mm diameter cell culture dishes, whose surface area is 6 × higher than that of p6 wells, are generally used, implying scaling the total amount of DNA to 15 µg. Additionally, double the amount of transgene-encoding plasmid is combined with packaging and envelope plasmids in a 2:1:1 molecule:molecule ratio to increase viral production [39]. According to their molecular weight, we used 7 μg of transgene-encoding plasmid (bicistronic pMX-GFP-Sox15, 6750 bp), 5.7 μg of packaging protein-encoding plasmid (pCMV-GAG-POL, 11 kbp) and 2.3 μg of envelope protein-encoding plasmid (pCMV-VSV, 6050 bp). We then followed our optimized CCD generation, maintaining the efficient CD:PEI:DNA ratio to 20:3:1, thus combining 45 µg of PEI with 300 µg of CDs to generate CCDs that were then mixed with DNA before cell transfection on HEK293T packing cells.

**Fig. 6:**
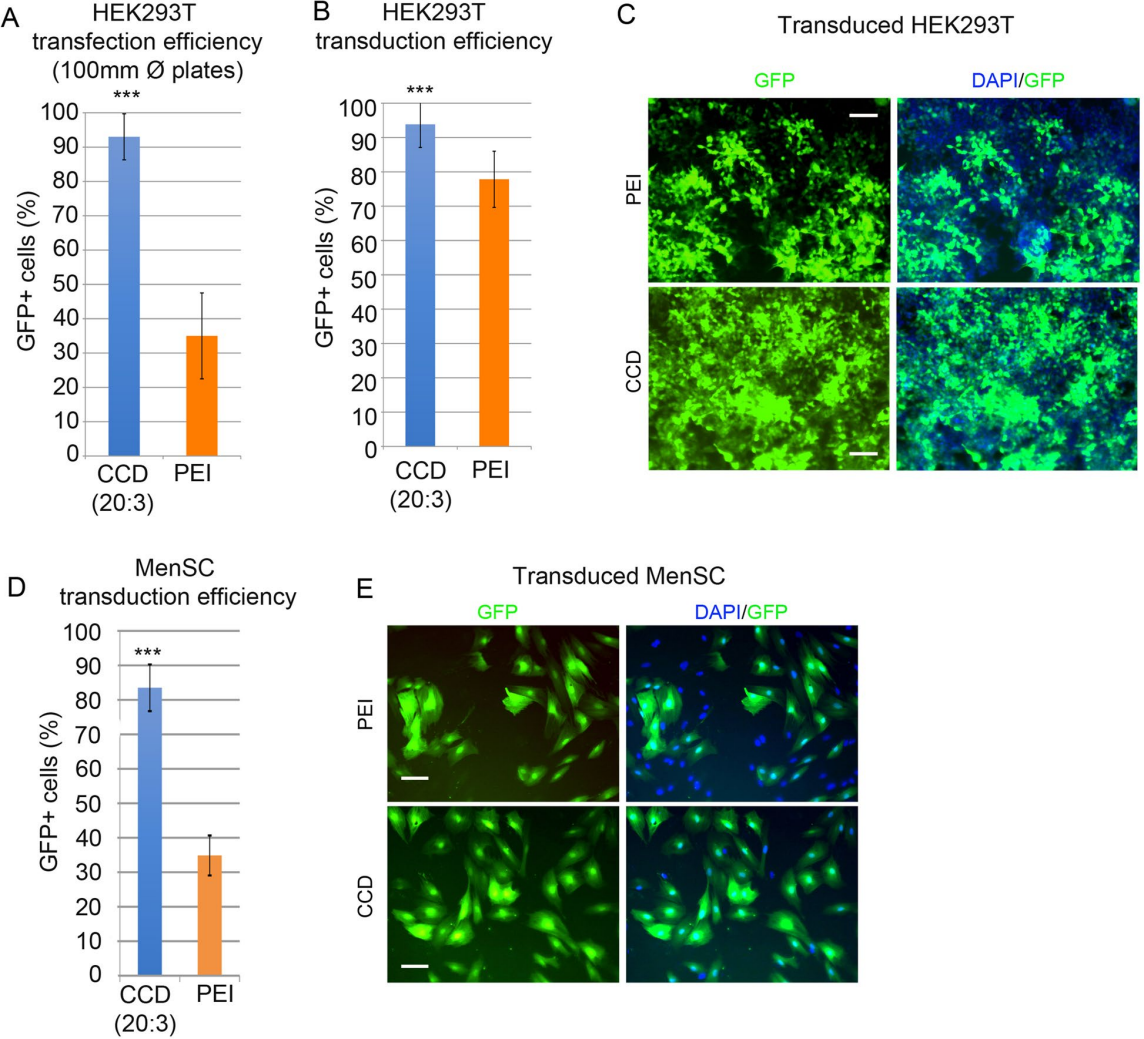
**A** Transfection efficiency measured as the % of GFP-positive cells analyzed by flow cytometry of HEK293T cells plated in 100 mm diameter dishes and transfected with CCDs generated by incubation of CDs with PEI at a 20:3 mass ratio and mixed with a fixed amount of DNA to obtain the optimized 20:3:1 mass ratio. The standard transfection using PEI at a 3:1 mass ratio with DNA was used as a control (orange) (*n* = 11, mean values ± SD). Student’s t-test was applied for statistical significance: ^***^ *P* < 0.001) **B** Viral transduction efficiency measured as the % of GFP-positive cells analyzed by flow cytometry of HEK293T cells after incubation with viral-containing supernatant secreted by HEK293T cells after three vector cotransfection using either CCD or PEI conditions (*n* = 9, mean values ± SD. Student’s t-test was applied for statistical significance: ^***^P < 0.001) **C** Representative fluorescence microscopy images of viral transduced HEK293T cells as in (**B**) (scale bar = 100 µm). **D** Viral transduction efficiency measured as the % of GFP-positive cells analyzed by flow cytometry of MenSC cells after incubation with viral-containing supernatant secreted by HEK293T cells after three vector cotransfection using either CCD or PEI conditions (*n* = 3, mean values ± SD. Student’s t-test was applied for statistical significance: ^***^P < 0.001) **E** Representative fluorescence microscopy images of viral transduced MenSC cells as in (**D**) (scale bar = 100 µm)

We first confirmed that CCDs significantly increased cell transfection efficiency after scaling up the assay, as shown by flow cytometry quantification of GFP-positive cells (Fig. 6A). Then, proper retroviral particle production was analyzed. For this, cell culture supernatant was recovered 24 h after transfection and was used to transduce cells. Retroviral particles containing the transgene constructor are able to infect target cells, integrate into their genome and express exogenous GFP and Sox15 genes. Viral supernatant coming from CCD transfection showed the highest transduction efficiency of both HEK293T (Fig. 6B,C) and MenSCs (Fig. 6D,E), measured as the % of GFP positive cells (Figs. 6B,D) and as the level of fluorescence intensity measured by flow cytometry (Supplementary Figure S2) indicating the highest production of viral particles after CCD transfection.

Effective internalization of DNA by cells is a crucial factor for the transfection process. Cell endocytosis is a complex process that has yet to be completely defined despite significant progress in the understanding of different endocytic routes [33]. The mechanism that allows the content of endocytosed vesicles to escape their degradation by late endosome and lysosome activity, which is the most common destiny of endocytosed vesicles, is not completely understood [33, 40]. If DNA does not escape from endosomes before reaching lysosomes, it is probably lost for gene expression, and the DNA in recycling endosomes is also most likely lost for the purposes of transfection. However, several reviews mention the possibility of vesicles shuttling between lysosomes and the Golgi or the ER, which would give the endocytosed DNA a last chance to escape degradation [40, 41]. In addition, it has been demonstrated that endosomal release of synthetic compounds such as DNA polyplexes can be facilitated via the covalent linkage of polyethylenimines to melittin analogs [42, 43] or through a proton sponge mechanism, a hypothesis describing that PEI/DNA complexes could promote proton and chloride influx into endosomes leading to disruption of endosomes and release of DNA into the cytosol [44], thus explaining the efficiency of our PEI-coated CCD-mediated transfection method that further enables gene expression of plasmid-encoded transgenes whose functionality was proven optimal even for demanding viral production of transfected cells.

The interaction of nanocarriers with plasma membrane is highly dependent on a myriad of factors including particle size, shape, surface structure, charge, lipophilicity, nature of nanocarrier and cell type involved in internalization [45, 46]. We hypothesize that the coating of CD with PEI generates CCD whose structure has a great impact on cell membrane interaction and/or internalization as the interaction of nanoparticles with cell membrane plays a crucial role in endocytosis and intracellular trafficking.

## Conclusion

Here, we present a simple and affordable method for efficient cell transfection through the preparation of CCDs by electrostatic binding of citric-based CDs prepared via a hydrothermal method with linear 25 kDa PEI using room temperature agitation. We present a biofunctional approach to estimate the optimal ratio of CD:PEI that can be easily modified and scaled up for specific transfection conditions. The CCDs effectively bound DNA into nanoparticles, increased cell viability over the standard PEI method and enabled functional expression of exogenous genes, thus escaping cell degradation. Cell transfection using CCDs increases over four times the standard of PEI polyplexes, reaching more than 90% of HEK293T cell transfection efficiency. The HEK293T cell line is a reference platform for the challenging large-scale production of viral-based products that require the most efficient transfection methods to achieve retroviral generation. Our described CCDs enable the successful production of retroviral particles encoding desired transgenes, confirming their suitability as a transfection method for biological applications and the manufacturing of scalable recombinant products.

### Supplementary Information

**Supplementary Material 1. **

## Data Availability

The datasets used and/or analysed during the current study are available from the corresponding author on reasonable request.
