# Supplementary material for: Efficient and scalable gene delivery method with easily generated cationic carbon dots
Source: Biol Proced Online. 2024 Mar 8;26:6. doi: 10.1186/s12575-024-00232-7 (PMC10921679; doi:10.1186/s12575-024-00232-7)
Supplement: Supplementary file 1 — Supplementary Material 1. [file 12575_2024_232_MOESM1_ESM.pdf]

Supplementary Information

Efficient and scalable gene delivery method with easily generated cationic carbon dots

Manuel Algarra[a] and Elena Gonzalez-Muñoz[b,c,*]

[a] INAMAT[2] - Institute for Advanced Materials and Mathematics. Department of Science. Public University of Navarra. 31006 Pamplona, Spain.

[b] Instituto de Investigación Biomédica de Málaga y Plataforma en Nanomedicina (IBIMA plataforma BIONAND) C/ Severo Ochoa, 35. Málaga. Spain.

[c] Departamento de Biología Celular Genética y Fisiología, Universidad de Málaga, 29071 Málaga, Spain

[*] corresponding author Elena Gonzalez Muñoz egonmu@uma.es

Supplementary Tables S1 and S2
Supplementary Figures S1 and S2

Supplementary Table S1. Transfection efficiency of CCD generated at different CD concentration and CD:PEI mass ratio using a constant PEI:DNA μg ratio of 3:1. Transfection efficiency is quantified as GFP% positive cells analyzed by flow cytometry. Relative cell viability (% RCV) measured by MTT assay as in Figure 5A and transfection yield (product of the cell % RCV and % transfection efficiency) are also shown.

CD (μg/L)	CCD Ratio CD:PEI	Transfection efficiency (%)	% RCV	Transfection Yield (%)
**100**	10:3	10 ± 2		
	20:3	60 ± 9	81.3 ± 4.6	48.8
	40:3	35 ± 6		
**50**	10:3	35 ± 5	89.2 ± 5.3	31.2
	**20:3**	**82 ± 10**	93.5 ± 7.6	**76.7**
	40:3	65 ± 7	91.6 ± 4.2	59.5
**25**	10:3	22 ± 3		
	20:3	53 ± 6	92.3 ± 6.8	48.9
	40:3	26 ± 4		

Supplementary Table S2. Transfection efficiency of CCD generated at a constant CD:PEI mass ratio of 20:3 using different amount of DNA. Transfection efficiency is quantified as GFP% positive cells analyzed by flow cytometry.

CD (μg/L)	CD:PEI	Transfection efficiency (%)	μg DNA (CD:PEI:DNA)	Transfection efficiency (%)
**50**	20:3	82	20:3:**0.2**	8 ±3
			20:3:**0.5**	33 ± 4
			20:3:**1**	**82 ± 10**
			20:3:**2**	78 ± 7
			20:3:**5**	70 ± 5

Supplementary Figure S1. (A,C,E) TEM images of a 50 g/L CCD suspension prepared as described by 3 hours of mixing CDs with PEI at a 20:3 optimized mass ratio (scale bar 50 nm), 2 hours (A), 8 hours (C) and 24 hours (E) after preparation. **(B,D,F)** Histogram frequency of DLS measurements of the mean hydrodynamic diameter (nm) found in CCD suspensions 2 hours (B), 8 hours (D) and 24 hours (F) after preparation. (G) Transfection efficiency measured as the % of GFP-positive cells analyzed by flow cytometry of HEK293T cells plated in p6 wells and transfected using CCDs 30 minutes, 2 hours, 8 hours and 24 hours after preparation. CCDs 30 minutes after preparation (light blue) and PEI at a 3:1 mass ratio with DNA (orange) are shown as transfection reference values (See Figure 3).

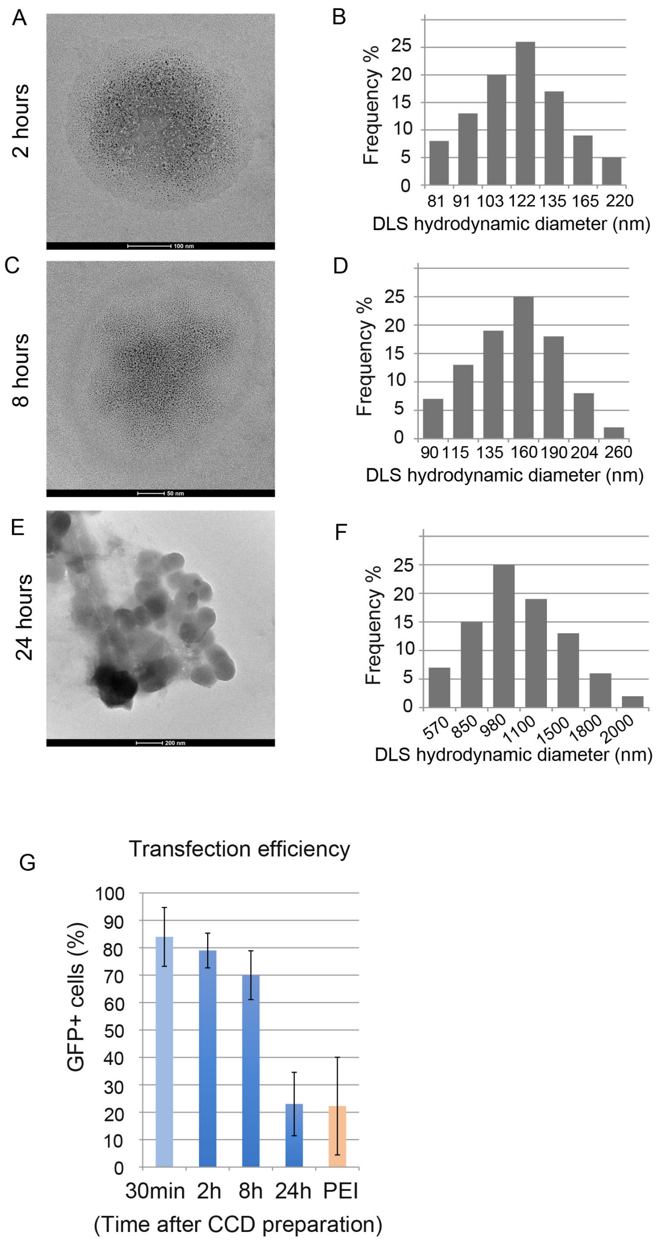

Supplementary Figure S2. Histograms of flow cytometry analysis of HEK293T (A) and MenSC (B) after transduction with viral-containing supernatant produced using transfection with either CCD or PEI conditions. Side table shows percentage of GFP positive cells as well as median and average mean fluorescence intensity values (related to Figure 6).

A

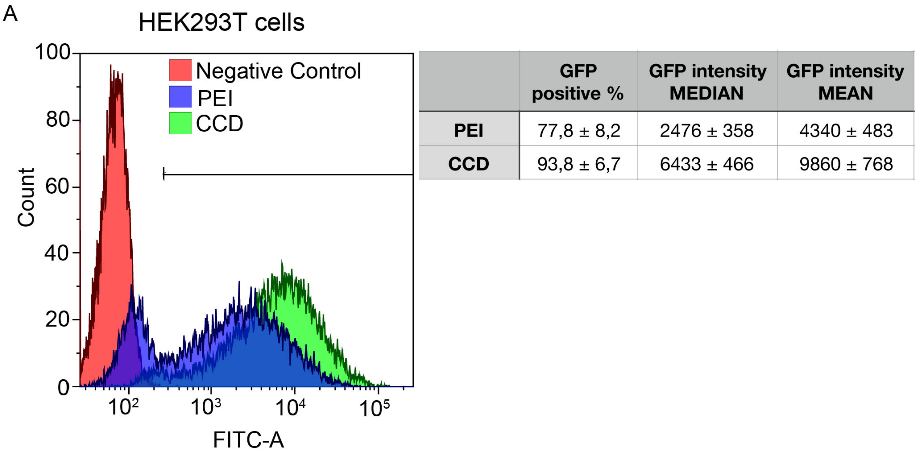

	GFP positive %	GFP intensity MEDIAN	GFP intensity MEAN
**PEI**	77,8 ± 8,2	2476 ± 358	4340 ± 483
**CCD**	93,8 ± 6,7	6433 ± 466	9860 ± 768

B

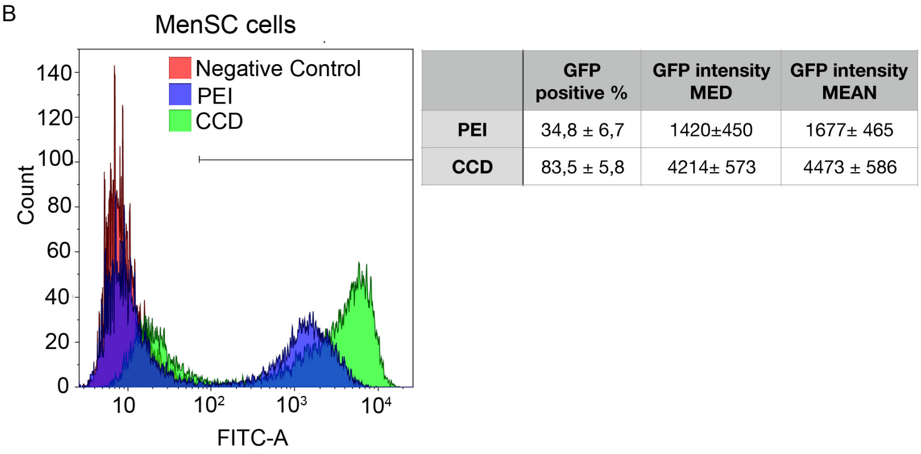

	GFP positive %	GFP intensity MED	GFP intensity MEAN
**PEI**	34,8 ± 6,7	1420±450	1677± 465
**CCD**	83,5 ± 5,8	4214± 573	4473 ± 586
